# Can jaw position affect the fine motor activity of the hand during writing?

**DOI:** 10.1002/brb3.1887

**Published:** 2020-10-21

**Authors:** Ahmad H. Alghadir, Hamayun Zafar, Zaheen A. Iqbal

**Affiliations:** ^1^ Department of Rehabilitation Sciences College of Applied Medical Sciences King Saud University Riyadh Saudi Arabia; ^2^ Department of Odontology Clinical Oral Physiology Faculty of Medicine Umea University Umea Sweden

**Keywords:** fine motor skills, handwriting, jaw position

## Abstract

**Background:**

Jaw and neck systems have been shown to be functionally related and changes in either system can modulate gross motor functions, such as posture control. It remains to be seen if any change in jaw position can affect fine motor skills. The objective of this study was to determine the effect of resting, open and clenched jaw positions on various handwriting parameters while standing on firm and unstable surfaces.

**Methods:**

Handwriting samples were collected from 36 healthy male participants (age, 15–35 years) using a digitizer tablet (WACOM Intuos 4) with noninking pen in the resting, open and clenched jaw positions while standing on firm and unstable surfaces. The measured handwriting parameters included duration, vertical size, horizontal size, absolute size, average absolute velocity, and absolute jerk. Recordings and analyses were performed using NeuroScript MovAlyzeR software.

**Results:**

All handwriting parameters varied among the resting, open, and clenched jaw positions on both the firm and unstable surfaces. However, based on statistical analyses, there were no significant differences in the handwriting parameters among three jaw positions on both surfaces (*p* > .05).

**Conclusion:**

This study revealed that all handwriting parameters varied among the resting, open, and clenched jaw positions on both the firm and unstable surfaces, showing that change in the jaw motor system may potentially affect the fine motor skills. However, on statistical analysis, there was no significant effect of 3 studied jaw positions on fine motor skills as seen on gross motor skills among healthy individuals.

## 1 INTRODUCTION

Handwriting is used to express thoughts in everyday life and has been shown to be associated with academic and social development among children (Kushki et al., 2011). It is an automated skill, which starts to develop at 3 years of age, and is completed by 8–9 years of age (Feder & Majnemer, 2007; Tseng & Chow, 2000; Tucha & Lange, 2004a). The biomechanics underlying the process of writing are very complex (Jones & Christensen, 1999). Handwriting is considered to be a perceptual‐motor skill in which signals are transferred from the brain to the peripheral level, involving various muscles and joints of the upper limb (Berninger, 1999; Teulings & Thomassen, 1989). Handwriting requires psychomotor activity involving visual‐motor coordination across opposite muscles and joint movements across various degrees of freedom (Rosenblum et al., 2010). Along with cognitive processes, various kinesthetic sensitivities are also involved in handwriting, including those related to pen control and appropriate force production (Berninger et al., 1991; Feder & Majnemer, 2007). These components are a result of fine motor control, which include in‐hand manipulation, bilateral integration, and motor planning (Cornhill & Case‐Smith, 1996). Due to the involvement of such processes, handwriting is highly sensitive to neurologic disturbances (Kushki et al., 2011).

Various studies have examined the nature and cause of handwriting difficulties, especially in children and patients with various neurological disorders (Caligiuri, Teulings, Dean, et al., 2009; Van Gemmert et al., 2001; Kushki et al., 2011). Such studies either analyzed the handwriting output on the basis of quality and rate or evaluated the kinetic and kinematic data associated with handwriting through digital tablets and sensors associated with the process of writing (Mavrogiorgou et al., 2001; Smits‐Engelsman & Van Galen, 1997; Werner & Rosenblum, 2004). Fatigue, mental as well as physical, has been shown to affect the handwriting of healthy individuals and of those with movement disorders (Van Gemmert et al., 2001; Provins & Magliaro, 1989). In forensic based analysis, even a subtle change in handwriting can cause problems in identification and verification. Forensic experts must rule out various conditions, such as fatigue, which may affect the pattern of writing, before rejecting an identity claim. In addition, the absence of gravitational force influences perceptual‐motor tasks, including writing and drawing, in astronauts (Clement et al., 2009; Lathan et al., 2000).

The jaw and neck regions have been shown to be anatomically, biomechanically, and neurologically linked to each other (Alghadir et al., 2015; Eriksson et al., 1998; Zafar et al., 2000). Jaw clenching has been shown to improve distal muscle strength and motor performance in various tasks, including sports activities (Alghadir et al., 2019; Cherry et al., 2010; Ebben, 2006). Furthermore, activation of the jaw sensory motor system through changes in its static (clenching) and dynamic (chewing) positions have been shown to increase body stability through modulation of body posture control mechanisms while standing on an unstable surface, in both presence and absence of vision (Alghadir et al., 2014; Alghadir et al., 2015; Zafar et al., 2020). Taken together, these studies have shown that the jaw and neck systems are functionally related and that changes in either system can modulate gross motor functions, such as posture control.

Although, there are various studies in the literature that show the effect of jaw positions on gross motor functions, to the best of our knowledge, no study has evaluated similar effects on fine motor functions, such as handwriting. This study was conducted to see the effect of resting, open, and clenched jaw positions on various handwriting parameters while standing on firm and unstable surface. We postulated that change in the jaw motor system may potentially change the fine motor activity of hand during writing.

## 2 METHODS

### 2.1 Participants

Thirty‐six healthy male participants (age, 15–35 years) were included in this study. Before the study, all participants were subjectively and objectively assessed for any balance or jaw disorders and were excluded if any disorder was found. Their height in centimeters and weight in kilograms were also noted. All participants were informed of the aims and procedures of the study and provided written informed consent. In the case of minor participants (age < 16 years), informed consent was obtained from the parents/legal guardians. This study was approved by the rehabilitation research review board for ethics according to the guidelines of the Declaration of Helsinki (reference no. KSU/RRC/026/02).

### 2.2 Apparatus and handwriting parameters

Hand writing samples were collected using a digitizer tablet (Intuos 4, Wacom) with noninking pen (Intuos non‐Inking Pen for Wacom digitizers), as used in previous studies (Caligiuri, Teulings, Dean, et al., 2009; Kushki et al., 2011; Mavrogiorgou et al., 2001; Tucha & Lange, 2004a). The sampling rate was 100 Hz. Recordings and analyses were performed using a dedicated script analysis software (MovAlyzeR, NeuroScript, LLC). Handwriting features were recorded for the complete writing pattern of a standardized phrase. Data on duration (time interval between the first and last samples in a stroke), vertical size (vertical amplitude difference between the beginning and end of a stroke), horizontal size (horizontal amplitude difference between the beginning and end of a stroke), absolute size (absolute size based on the amplitude of a stroke or segment), average absolute velocity (average absolute velocity across all samples of a stroke or segment), and absolute jerk (third derivative of position or change in acceleration due to change in force produced to propel the pen over the tablet) were recorded (Caligiuri, Teulings, Dean, et al., 2009; Guinet & Kandel, 2010; Maarse & Thomassen, 1983; Teulings et al., 1997).

### 2.3 Procedure

Participants were asked to stand comfortably with the noninking pen in their dominant hand and the tablet in their other hand. They were asked to practice writing on the tablet to become familiar with the recording protocol. Participants were asked to write “happy new year” in their own normal cursive handwriting in three test jaw positions while standing on firm and unstable (a 50 × 50 × 15‐cm foam block) surfaces. In the first jaw position, no instructions were given (resting jaw). In the second jaw position, participants were asked to open their jaw throughout the test (open jaw). In the third jaw position, participants were asked to clench their teeth throughout the test (clenched jaw) (Ahmad Alghadir et al., 2017; Alghadir et al., 2015; Zafar et al., 2017; Zafar et al., 2019). Three trials for each condition were recorded in a random order, and their mean values were used in the data analyses. The entire process of data collection took an average of 15 min.

### 2.4 Statistical analysis

Data were analyzed using Graph‐Pad Instat 3.0 (GraphPad Software). Descriptive statistics were presented as mean and standard deviation (*SD*). Repeated measures analysis of variance (ANOVA) was used to compare the differences in the handwriting parameters among the three test positions. The null hypothesis was rejected at a significance level of .05.

## 3 RESULTS

### 3.1 Demographic characteristics

The mean age of the participants was 23.3 years (*SD*, 2.75). The mean height was 174 cm (*SD*, 5.5), and the mean weight was 75.2 kg (*SD*, 8.8). All participants, except one, were right‐handed.

### 3.2 Comparison of handwriting parameters in the three test positions on firm and unstable surfaces

Table 1 shows that all handwriting parameters varied among the resting, open, and clenched jaw positions on both, the firm and unstable surfaces. Compared to the resting and open jaw positions, a decrease in all parameters values was noted in the clenched jaw position on the firm surface. On the unstable surface, there was an increase in the vertical, horizontal, and absolute size and in absolute jerk were noted in the clenched jaw position compared with the resting and open jaw positions. There were no significant differences in the handwriting parameters among the resting, open, and clenched jaw conditions on both the firm and unstable surfaces (*p* > .05).

**TABLE 1:** Handwriting features of 36 healthy male participants in three test positions while standing on firm and unstable surfaces

Feature	Firm surface			Unstable surface		
	Resting jaw	Open jaw	Clenched jaw	Resting jaw	Open jaw	Clenched jaw
Duration, s	8.26 (2.15)	7.81 (1.84)	7.64 (2.12)	7.42 (2.23)	7.89 (1.93)	7.64 (1.97)
Vertical size, cm	2.41 (5.72)	3.09 (5.40)	2.26 (5.17)	2.03 (5.49)	2.17 (5.89)	2.47 (5.33)
Horizontal size, cm	20.30 (7.49)	19.82 (6.95)	19.14 (7.25)	19.34 (6.84)	21.75 (7.02)	20.68 (7.06)
Absolute size, cm	21.34 (7.24)	20.93 (6.58)	20.22 (6.77)	20.41 (6.52)	22.82 (6.51)	21.73 (6.58)
Average absolute velocity, cm/s	10.00 (3.12)	10.31 (3.06)	10.47 (3.09)	11.12 (3.30)	10.77 (3.10)	10.99 (3.39)
Absolute jerk, cm/s^3^	13,673.63 (5,193.20)	13,549.36 (4,696.80)	13,437.47 (4,111.50)	14,390.02 (4,379.00)	15,065.33 (5,356.60)	14,548.66 (5,045.70)

Note

All data are presented as mean (*SD*).

### 3.3 Comparison of handwriting parameters in the three test positions between firm and unstable surfaces

There were no significant differences in the handwriting parameters among the resting, open, and clenched jaw positions between the firm and unstable surfaces (*p* > .05).

## 4 DISCUSSION

This study evaluated the effect of different jaw positions on the fine motor skill of handwriting while standing on firm and unstable surfaces. We found that all handwriting parameters varied among the three jaw positions on both the firm and unstable surfaces, showing that change in the jaw motor system may potentially affect the fine motor skills. However, based on statistical analysis, there were no significant differences in the handwriting parameters among the three jaw positions on both surfaces.

The changes in the features of handwriting may be due to different strategies of the motor system to control the handwriting pattern. The motor system compensates for disturbed input by employing a biomechanical strategy that involves limb stiffness (van Den Heuvel et al., 1998). Increased stiffness in the limb muscles can decrease movement speed while writing (Vangalen & Schomaker, 1992), which may lead to a decrease in the absolute and, specifically, vertical size of the word, as observed in our results.

The role of the standing surface in maintaining body stability has been established, as standing on an unstable surface poses a significant challenge to postural control (Hatton et al., 2011; Mohapatra et al., 2014). While standing on an unstable surface, irrespective of jaw position, we found that there was a decrease in duration and horizontal, vertical, and absolute word size, as well as an increase in average absolute velocity and absolute jerk (Table 1). This could be explained by the following phenomenon: When the participant would try to complete his writing task quickly, to compensate for postural instability and prevent falling, he would automatically increase his writing speed and decrease the word size. Thus, our results show that there are changes in some features of handwriting when participants write while standing on an unstable surface, where the body is in a more challenging situation, indicating that the jaw motor system has the capacity of affect fine motor activity.

Legibility and speed are the two most important elements of handwriting (Feder & Majnemer, 2007; Kushki et al., 2011). Handwriting difficulties include poor quality of letter formation, alignment, sizing, and spacing of letters (Feder & Majnemer, 2007). Body posture affects the quality of handwriting (Tseng & Cermak, 1993). For example, forward bending while writing is associated with higher degrees of muscle tension, leading to poor handwriting quality (Parush et al., 1998). Our results show that changes in jaw position have potential stimulating effects on the psychomotor performance of skilled actions performed in everyday life, such as handwriting, which do not require conscious control or additional attention.

During the clenched jaw position when standing on an unstable surface, we observed an increase in the vertical, horizontal, and absolute size and in absolute jerk and a decrease in the average absolute velocity compared with writing while standing on a firm surface. Handwriting involves high and low order processes that are responsible for its composition and transcription (Graham & Harris, 2000). While the higher order processes include activation of intensions, semantic retrieval, and syntactic construction, the low order processes involve allograph selection, size control, and muscle adjustment (Vangalen, 1991). The clenched jaw position affects various descending pathways from the central nervous system through various mechanisms via an increase in distal muscle strength, neck muscle endurance, and postural stability (Alghadir et al., 2015; Ebben et al., 2010; Zafar et al., 2019). Our results also show that besides the effect on gross motor functions, jaw positions also have an effect on the fine motor functions.

The tablet used in this study for data recording and analysis has been extensively used in the previous studies to report the kinetics and kinematics of handwriting among different healthy and diseased participants (Caligiuri, Teulings, Niculescu, et al., 2009; Caligiuri, Teulings, Dean, et al., 2009; Kushki et al., 2011; Mavrogiorgou et al., 2001; Teulings et al., 1997; Tucha & Lange, 2004a, 2004b). The nonsignificant results in this study may be attributed to the limited number of healthy participants included. Thus, similar studies should be repeated among participants with equal gender representation and those with learning difficulties and movement disorders to further clarify whether modification of jaw position affects fine motor skills using a more sensitive outcome measures to detect the subtle changes in the quality of handwriting.

## 5 CONCLUSIONS

This study revealed that all handwriting parameters varied among the resting, open, and clenched jaw positions on both the firm and unstable surfaces, showing that change in the jaw motor system may potentially affect the fine motor skills. However, on statistical analysis, there was no significant effect of 3 studied jaw positions on fine motor skills as seen on gross motor skills among healthy individuals.

## CONFLICT OF INTEREST

The authors declare that they have no competing interests.

## AUTHOR CONTRIBUTIONS

Research idea and design were proposed by HZ and ZAI. Review of literature was done by AHA, HZ, and ZI. Data collection and analysis were executed by ZI and HZ. Manuscript preparation and submission were done by AA and ZI.

## ETHICAL APPROVAL

All participants were informed of the aims and procedures of the study and provided written informed consent. In the case of minor participants (age < 16 years), informed consent was obtained from the parents/legal guardians. This study was approved by the rehabilitation research review board for ethics according to the guidelines of the Declaration of Helsinki (reference no. KSU/RRC/026/02).

## Data Availability

The datasets used in this study are available from the corresponding author on request.
